# Neurodevelopmental disorder-associated *ZBTB20* gene variants affect dendritic and synaptic structure

**DOI:** 10.1371/journal.pone.0203760

**Published:** 2018-10-03

**Authors:** Kelly A. Jones, Yue Luo, Lynn Dukes-Rimsky, Deepak P. Srivastava, Richa Koul-Tewari, Theron A. Russell, Lauren P. Shapiro, Anand K. Srivastava, Peter Penzes

**Affiliations:** 1 Department of Physiology, Northwestern University Feinberg School of Medicine, Chicago, Illinois, United States of America; 2 J.C. Self Research Institute of Human Genetics, Greenwood Genetic Center, Greenwood, South Carolina, United States of America; 3 Department of Genetics and Biochemistry, Clemson University, Clemson, South Carolina, United States of America; 4 Department of Psychiatry and Behavioral Sciences, Northwestern University Feinberg School of Medicine, Chicago, Illinois, United States of America; University of South Alabama, UNITED STATES

## Abstract

Dendritic spine morphology and dendritic arborization are key determinants of neuronal connectivity and play critical roles in learning, memory and behavior function. Recently, defects of ZBTB20, a BTB and zinc finger domain containing transcriptional repressor, have been implicated in a wide range of neurodevelopmental disorders, including intellectual disability and autism. Here we show distinct effects of expression of two major isoforms, long and short, of ZBTB20, and its neurodevelopmental disorder-linked variants, on dendritic architecture of cultured rat cortical pyramidal neurons. The N-terminal of ZBTB20 showed a role in regulating dendritic spine morphology. Two ZBTB20 single nucleotide variants, located at the N-terminal and central regions of the protein and potentially conferring autism risk, altered dendritic spine morphology. In contrast, a single nucleotide variant identified in patients with intellectual disability and located at the C-terminus of ZBTB20 affected dendritic arborization and dendritic length but had no effect on dendritic spine morphology. Furthermore, truncation of the extreme C-terminus of ZBTB20 caused spine and dendritic morphological changes that were similar but distinct from those caused by the C-terminal variant. Taken together, our study suggests ZBTB20’s role in dendritic and synaptic structure and provide possible mechanisms of its effect in neurodevelopmental disorders.

## Introduction

Dendritic branches and spines are key regulators of neuronal function and essential for the formation and plasticity of neuronal circuits [1, 2]. Many of the few identified genes associated with neurodevelopmental disorders (NDDs), such as autism spectrum disorder (ASD) and intellectual disability (ID) have been found to affect both structures’ number and morphology and are consequently linked to the control of neuronal connectivity [3–7]. A network-based analysis of genes affected by rare *de novo* copy number variants (CNVs) in autism suggested that perturbed dendritic morphogenesis and synaptogenesis are key causes of autism [8]. Several studies suggest that the changes in neuronal gene expression controlled by selective expression of transcription factors (TFs) affect the formation of dendritic spines and synapses (reviewed in [9]).

**Fig 1:**
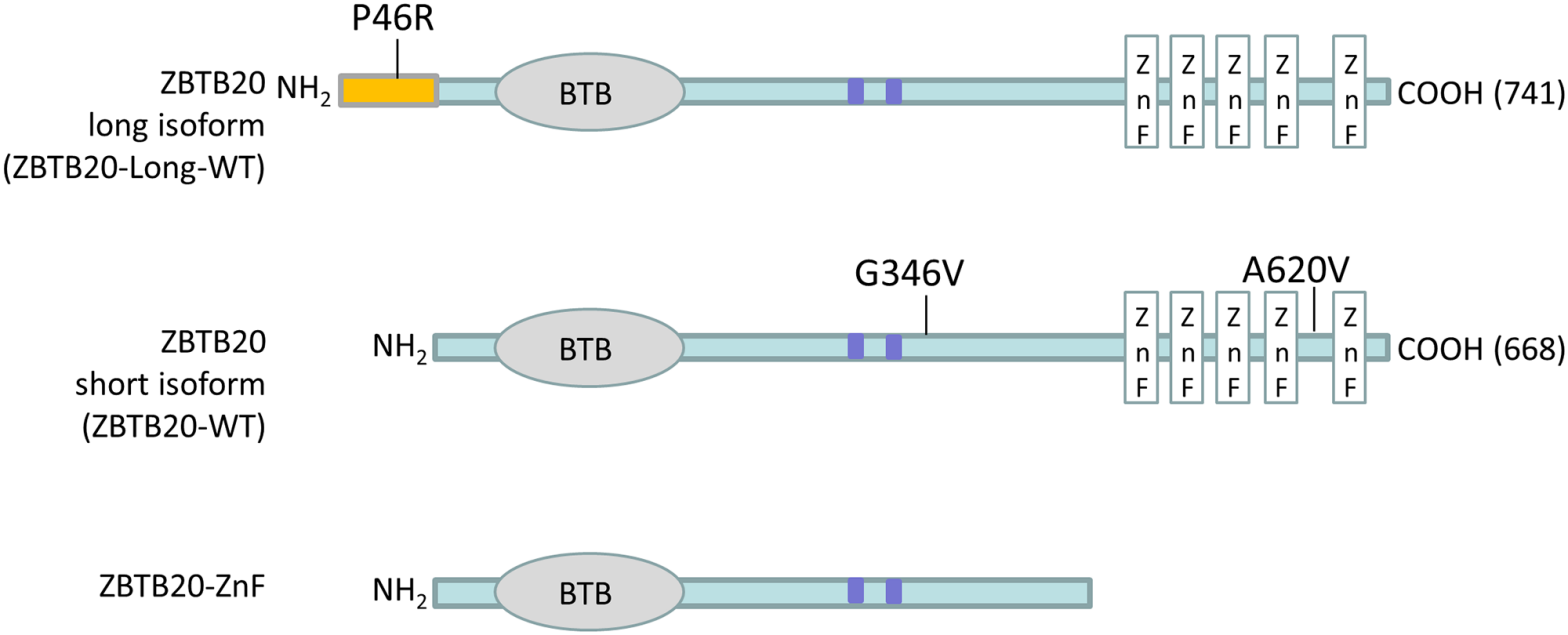
Schematic representation of ZBTB20. The BTB-domain (BTB), predicted sumoylation sites (blue boxes) in the central linker region, and five zinc fingers (ZnFs) in the short and long isoforms of ZBTB20, along with a region specific to the long isoform that extends from amino acid 1 to 73, are indicated. Previously identified ZBTB20 variants studied here are shown.

ZBTB20 is a member of the Broad complex, Tramtrack, and Bric-à-brac (BTB) and poxvirus and zinc finger (POZ) family of transcriptional repressors [10], and contains a BTB domain at the N-terminal, five C2H2-type zinc fingers at the C-terminal, and two predicted sumoylation sites located in the linker region between the BTB and zinc finger motifs (Fig 1). The human gene is expressed in variable amounts in different tissues including fetal brain. The mouse *Zbtb20* gene is highly expressed in the forebrain, particularly in the cortex and hippocampus, regions involved in learning, memory and behavioral function [10, 11].

Defects of *ZBTB20* have been implicated in a wide range of NDDs, including chromosome 3q13.31 microdeletion and microduplication syndromes, Primrose syndrome, ID, and in ASD [12–17]. Compared to the 3q13.31 microdeletion syndrome, a more severe phenotype has been noted in Primrose syndrome patients, including macrocephaly, ID, disturbed behavior, dysmorphic facial features, increased postnatal growth, diabetes, deafness, progressive muscle wasting, and calcified pinnae. Interestingly, all reported Primrose syndrome mutations were found to be located within the C-terminus zinc finger domain, whereas variants found to confer autism risk were reportedly located at the N-terminus. It has been suggested that dosage imbalance of *ZBTB20* contributes to this wide range of neurodevelopmental and neurobehavioral disorders (Fig 1). Transgenic mice with ectopic expression of *Zbtb20* show defects in cortical lamination and behavioral abnormalities [18]. Furthermore, the targeted deletion of *Zbtb20* in mice results in severe impairment in CA3 and dentate gyrus projections along with reduced hippocampus size [18], and downregulation of *Zbtb20* expression by RNA interference impairs the normal maturation of hippocampal CA1 pyramidal neurons, resulting in reduced apical dendritic arborization [19].

The molecular mechanism of ZBTB20’s physiological function in brain is largely unknown. However, ZBTB20 has been shown to regulate expression of many neuronal genes associated with processes such as neuronal development and morphogenesis, axogenesis, and synaptic transmission, including genes associated with NDDs such as *Cntn4*, *Gad1*, *Nrxn1*, *Nrxn3*, *Scn2a*, and *Snap25* [15]. Here we show that ZBTB20 isoforms, as well ZBTB20 variants affecting different functional domains of the protein, exhibit distinct effects on dendritic structure and dendritic spine morphology of cultured rat pyramidal neurons. Our findings suggest a potential role for ZBTB20 in neuronal structure and connectivity, and that genetic alterations in *ZBTB20* contribute to a spectrum of neurodevelopmental and neurobehavioral disorders. The findings also provide potential clues to the molecular mechanisms related to phenotypic variability associated with ZBTB20 variants.

## Results

### ZBTB20 isoforms induce distinct changes in dendritic spines

The *ZBTB20* gene encodes two major protein isoforms: the long 741-amino acid isoform and the short 668-amino acid isoform, which lacks the N-terminal 73 amino acids of the long isoform (Fig 1). Studies in mice have shown that both isoforms of Zbtb20 dimerize via the N-terminal regions containing the BTB domain and show overlapping expression patterns in the brain [18–22]. ZBTB20 has been shown to play a role in regulating pyramidal neuron morphology and development and hippocampal connectivity [18, 19]. Furthermore, alterations in synapse structure and function have been implicated in ID and ASD [3–7]. Members of the BTB/POZ-ZF family of transcription factors, in particular, have been shown to regulate dendritic morphology [23, 24]; thus, we sought to examine the effect of human ZBTB20 expression on synaptic and dendritic structure.

**Fig 2:**
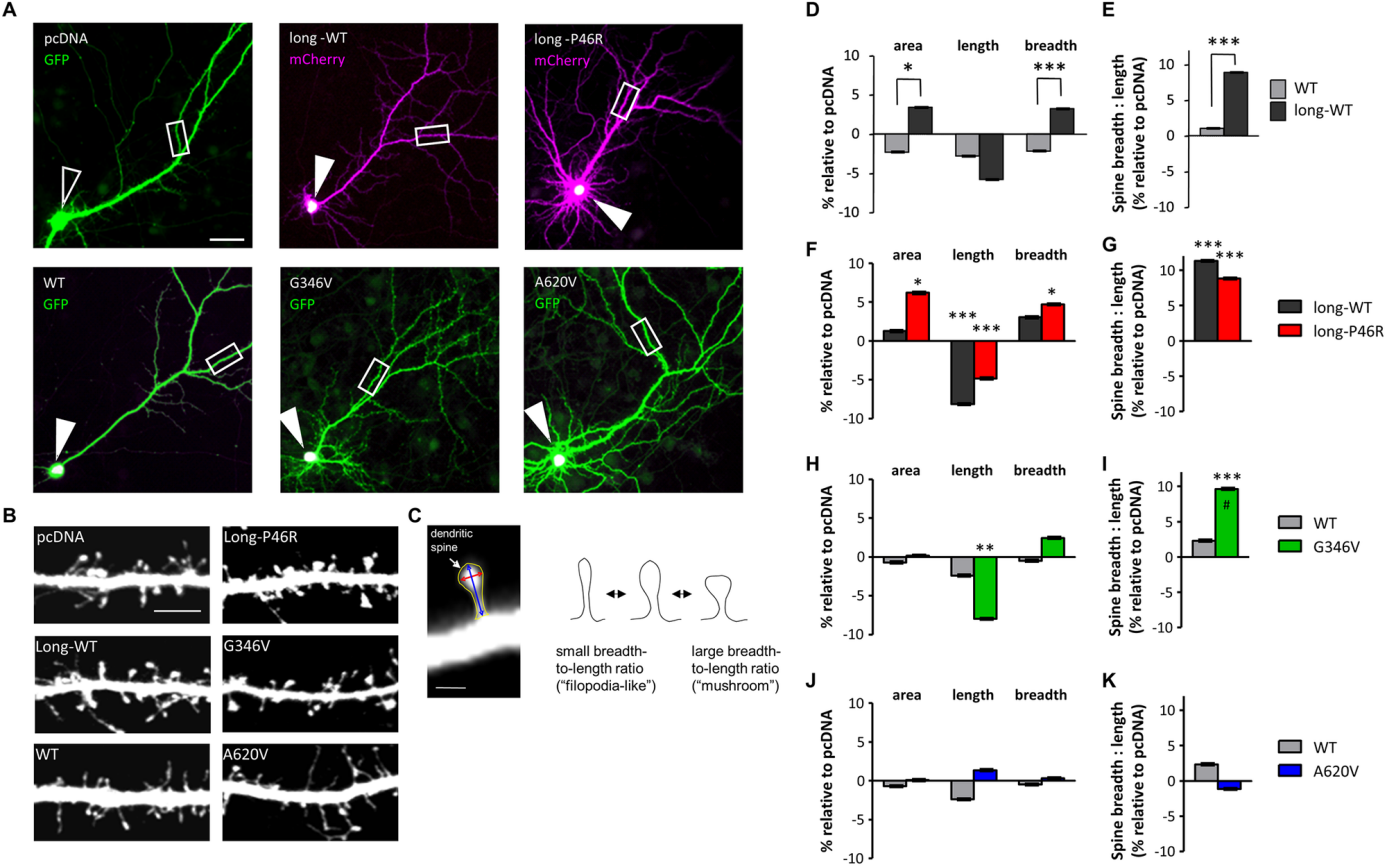
Effect of ZBTB20 and its variants on dendritic spine morphology. (**A**) Overexpressed ZBTB20 protein localized to the nucleus. Low magnification (10x) images of neurons overexpressing GFP + control vector (pcDNA), mCherry + ZBTB20-long-WT (GFP-tagged), mCherry + GFP-ZBTB20-long-P46R (GFP-tagged), GFP + ZBTB20-WT (V5-tagged), GFP + ZBTB20-G346V (V5-tagged), or GFP + ZBTB20-A620V (V5-tagged). Expression of control vector shows no V5 signal (open arrowhead), while overexpression of ZBTB20 constructs shows localization of ZBTB20 signal to the soma, presumably the nucleus (closed arrowheads; white indicates overlay of green and magenta channels). White boxes indicate regions of dendrites shown at higher magnification in Fig 3B. Scale bar, 40 μm. **(B**) Representative images of dendritic segments from forebrain pyramidal neurons (DIV 26–28) overexpressing the indicated ZBTB20 constructs. Scale bar, 5 μm (see also Fig 3). (**C**) Dendritic spines of pyramidal neurons were analyzed in MetaMorph for their cross-sectional area (yellow outline), length (blue arrow), and breadth (red). The breadth of each spine divided by its length gives its breadth-to-length ratio and indicates the spine’s shape independent of its size. Smaller breadth-to-length ratios indicate a thin, “filopodia-like” morphology, whereas larger breadth-to-length ratios indicate a short but broad, “mushroom-like” morphology. Scale bar, 1 μm. Dendritic spine areas, lengths, and breadths (**D, F, H, J**) and spine breadth-to-length ratios (**E, G, I, K**) of neurons expressing indicated ZBTB20 constructs [ZBTB20-WT (N = 16), ZBTB20-long-WT (N = 16), ZBTB20-long-P46R (N = 15), ZBTB20-G346V (N = 14), or ZBTB20-A620V (N = 18)]. All spine parameters are shown relative to control pcDNA vector. Data represented as means ± s.e.m. *P < 0.05; **P < 0.01; ***P < 0.001; #*P* < 0.001, different from WT. Student’s t test, point variant compared to WT counterpart.

We co-expressed short-wildtype (WT), and long-WT encoding constructs, with eGFP or mCherry to fill the transfected neurons, in cultured cortical pyramidal neurons, looked for their subcellular localization (Fig 2A), and performed morphometric analysis of dendritic spines (Fig 2B and 2C). Both isoforms were localized to a concentrated region of the soma, indicating their common subcellular localization (Fig 2A; white indicates overlay of green and magenta channels). However, expression of ZBTB20-long-WT increased spine area (*P* < 0.01), breadth (*P* < 0.0001), and breadth-to-length ratio, a measure of dendritic spine shapes independent of their areas (*P* < 0.0001), compared to ZBTB20-WT or the empty pcDNA vector control, without significantly changing spine length (Fig 2B, 2D and 2E; raw data in S1 Table). Expression of the shorter ZBTB20-WT did not significantly affect these parameters (Fig 2B, 2D and 2E), suggesting a role for the extreme N-terminus in regulating dendritic spine morphology.

### Effects of NDD-associated ZBTB20 variants on dendritic spines and dendritic arborization

Next, we examined the effect of three ZBTB20 variants putatively implicated in autism risk (P46R, G346V) and in patients with ID (A620V) [16, 17]. Similarly to wildtype ZBTB20, all three variants were localized to the soma, indicating that these nonsynonymous variants had no effect on the subcellular localization (Fig 2A). However, the ZBTB20-long-P46R variant, carrying an altered residue located within the N-terminal long isoform specific region (Fig 1), caused an abnormal spine enlargement resulting from a significant decrease in spine length (*P* < 0.001), and significant increases in spine area (*P* < 0.05), spine breadth (*P* < 0.05), and spine breadth-to-length ratio (*P* < 0.001) (Fig 2F and 2G). These changes were significantly different than its long-WT counterpart. The G346V variant, located in the central linker region near the ZBTB20 sumoylation site, caused a significant increase in spine breadth-to-length ratio (*P* < 0.001) compared to both pcDNA and ZBTB20-WT (Fig 2I) and a significant decrease in spine length compared to pcDNA (*P* < 0.01) (Fig 2H). Interestingly, the A620V variant, located at the C-terminal of ZBTB20, had no effect on dendritic spine morphology (Fig 2J and 2K). None of the variants had a significant effect on dendritic spine linear density (S1 Fig). Taken together, these data showed that ZBTB20 expression promoted short, broad, “mushroom-like” spine morphologies. Neurons overexpressing either P46R or G346V ZBTB20 variants produced distinct effects, and P46R also induced abnormal spine enlargement. These findings further emphasize importance of the N-terminus of ZBTB20 in regulation of dendritic spine morphology.

**Fig 3:**
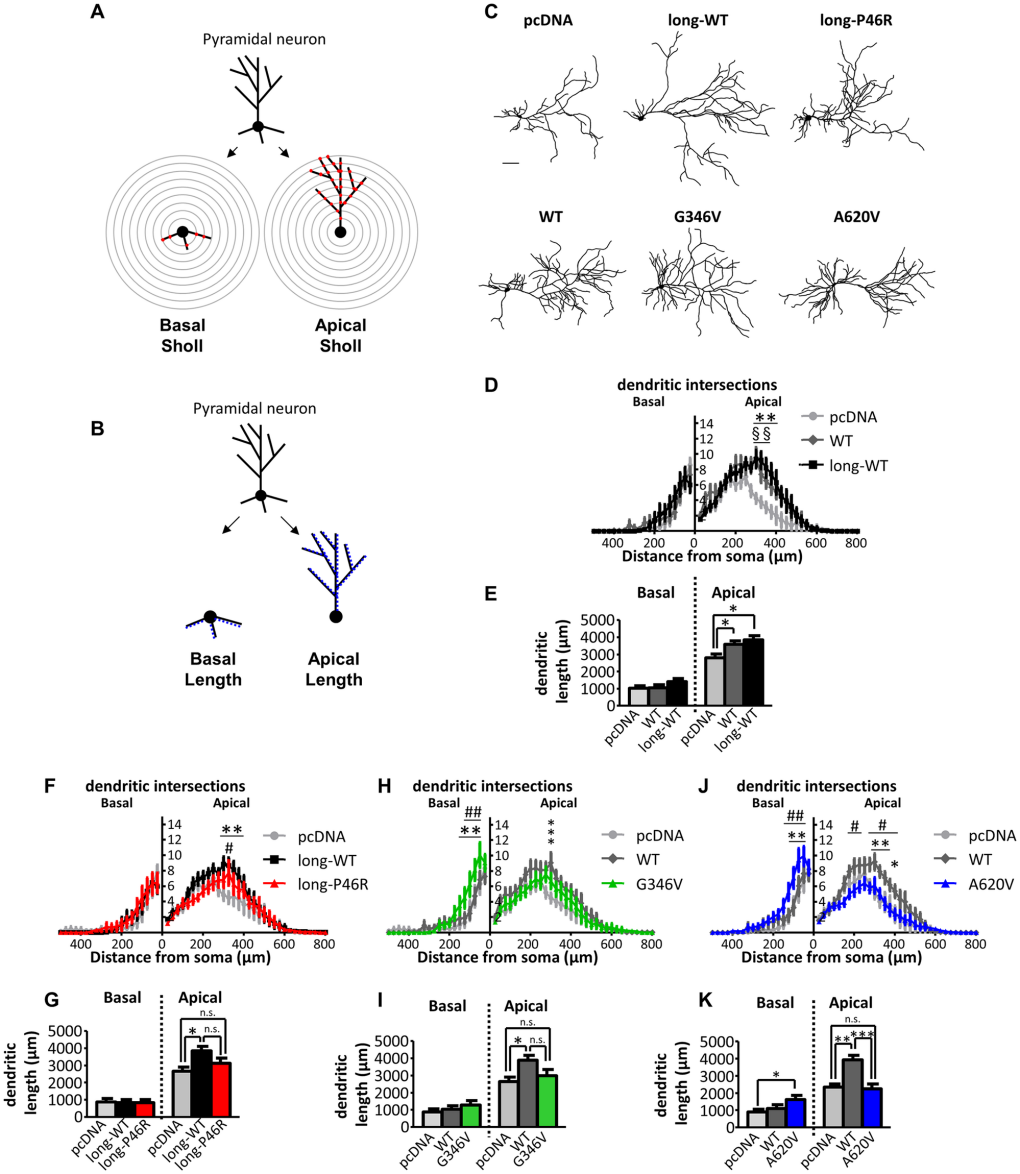
Effect of ZBTB20 and its variants on dendritic structure of pyramidal neurons. Sholl analysis (**A**) and dendritic length measurements (**B**) to assess dendritic complexity of ZBTB20-expressing neurons. Binarized traces of the complete dendritic arbor of each neuron were divided into apical and basal compartments. Apical and basal dendritic arbors were then measured separately via Sholl analysis (# of intersections of the dendritic arbor with concentric circles of increasing radii centered around the soma) and dendritic length (sum of lengths of all branches per dendritic compartment). (**C**) Representative tracings of cortical neurons (DIV 26–28) overexpressing indicated ZBTB20 constructs. Scale bar, 100 μm. Sholl analysis (**D**, **F**, **H, J**) and length measurement (**E**, **G**, **I, K**) of neurons expressing ZBTB20-WT (N = 22) or ZBTB20-long-WT (N = 16) (**D, E**), ZBTB20-long-P46R (N = 15) (**F, G**), ZBTB20-G346V (N = 17) (**H, I**), or ZBTB20-A620V (N = 18) (**J, K**) compared to pcDNA (N = 23) and wildtype counterparts. (**D, E**), *, WT compared to pcDNA; §, long-WT compared to pcDNA. *P < 0.05, ** or §§ P < 0.01. (**F-K**), *, WT or long-WT compared to pcDNA; #, point variant compared to WT or long-WT; n.s., not significant. *P < 0.05, **P < 0.01, ***P < 0.001. Data represented as means ± s.e.m. Scale bar, 100 μm (c). Sholl analysis: mixed model ANOVA (condition X distance from soma); length and spine parameters: one-way ANOVA.

Dendritic arborization plays a critical role in neuronal circuit formation. Thus, we next examined the effect of expression of ZBTB20 and its variants on dendritic structure of cortical pyramidal neurons (Fig 3). Sholl analysis and length measurements of the apical and basal dendritic compartments (Fig 3A, 3B and 3C) revealed that expression of both ZBTB20-long-WT and ZBTB20-WT, significantly increased apical arborization 300–400 μm from the soma (long-WT: *P* < 0.01; WT: *P* < 0.001) (Fig 3D), as well as apical dendritic length (long-WT: *P* < 0.05; WT: *P* < 0.05) (Fig 3E), to similar degrees, without altering basal arborization or basal length, compared to pcDNA control.

Examination of ZBTB20 variants revealed distinct effects on the dendritic arbor. ZBTB20-long-P46R did not significantly alter dendritic arborization or dendritic length compared to ZBTB20-long-WT (Fig 3C, 3F and 3G). ZBTB20-G346V had no effect on apical arborization, apical or basal dendritic length compared to ZBTB20-WT (Fig 3C, 3H and 3I), but increased basal dendrite arborization 50–100 μm from the soma (*P* < 0.0001; Fig 3H). In contrast to these autism-risk ZBTB20 variants, expression of A620V led to neurons with reduced apical arborization between 300–400 μm from the soma compared to ZBTB20-WT-expressing neurons (*P* < 0.05) and enhanced basal arborization 75–125 μm from the soma compared to ZBTB20-WT (*P* < 0.01) (Fig 3J). ZBTB20-A620V also reduced apical dendritic length compared to ZBTB20-WT (*P* < 0.0001) (Fig 3K). In summary, the most C-terminal variant in ZBTB20 (A620V) altered both basal and apical dendritic arborization, while the N-terminal variants (P46R and G346V) had limited effects on dendritic complexity. These data also suggest that the N-terminus does not determine effects of ZBTB20 on apical arborization.

### Effect of C-terminus of ZBTB20 on synaptic and dendritic structure

**Fig 4:**
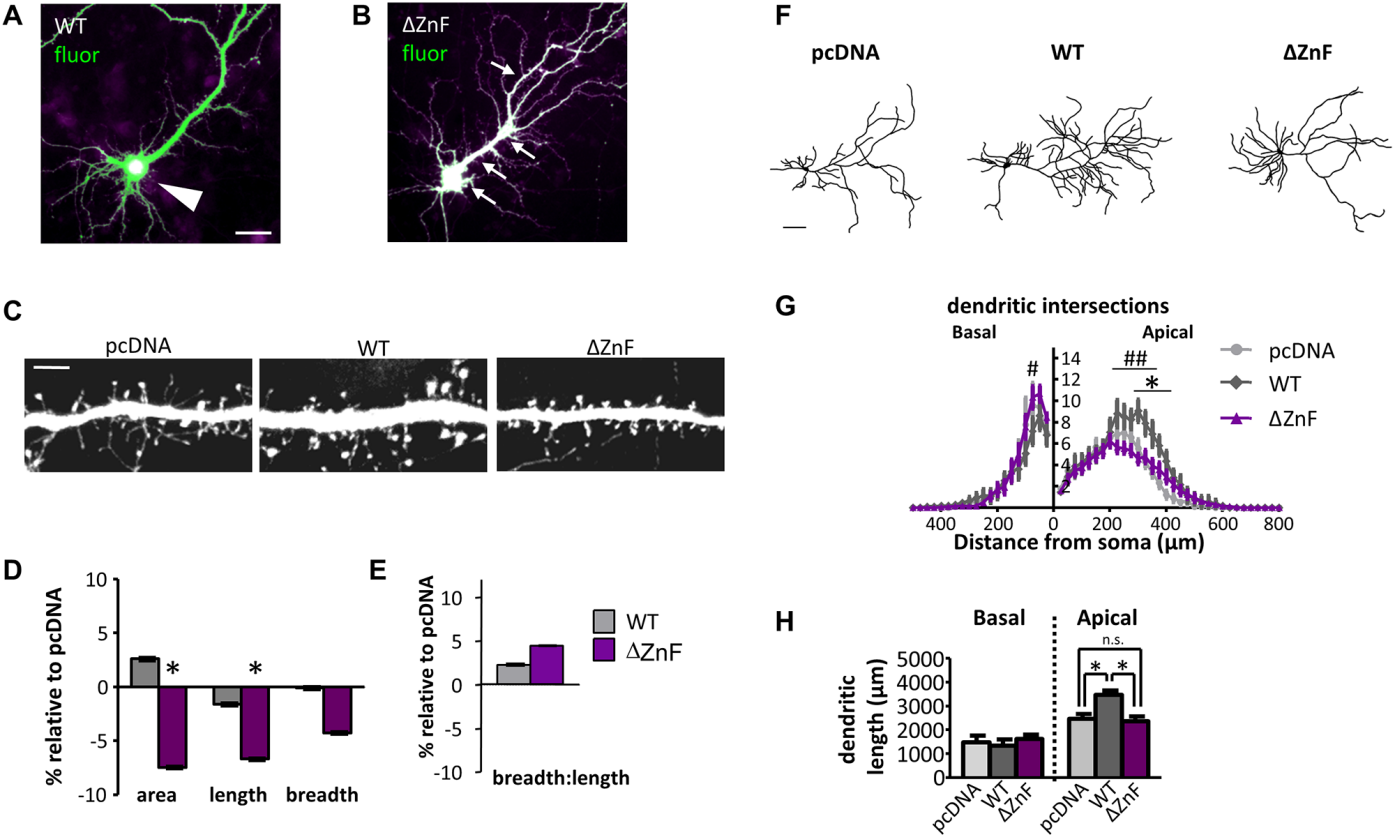
The C-terminal of ZBTB20 differentially regulates pyramidal neuron structure. (**A**) ZBTB20-WT localizes to soma (white arrowhead), but (**B**) ZBTB20-ΔZnF is unrestricted (arrows; white indicates overlay of green and magenta channels). **(C**) Representative images of dendritic spines from neurons (DIV 26–28) overexpressing the indicated constructs. (**D, E**) Spine areas, lengths, breadths, and breadth-to-length ratio of ZBTB20-ΔZnF (N = 9) compared to ZBTB20-WT (N = 8). Data shown as mean ± s.e.m., relative to the control vector (pcDNA). *P < 0.05 compared to pcDNA (N = 9). Scale bars, 25 μm (**A, B**), 5 μm (**C**). (**F**) Representative tracings of neurons overexpressing the indicated constructs. Sholl analysis (**G**) and dendrite length (**H**) of neurons expressing ZBTB20-WT (N = 11) or ZBTB20-ΔZnF (N = 23). *, WT compared to pcDNA (N = 9); #, ΔZnF compared to WT. *P < 0.05. Data represented as means ± s.e.m. Scale bar, 100 μm.

The two ZBTB20 isoforms and the A620V variant located in the C-terminus induced distinct cellular phenotypes in pyramidal neurons, suggesting that altering different subdomains of ZBTB20 may induce distinct structural alterations at the cellular level. Thus, to test the function of the C-terminus, we generated a variant lacking the zinc fingers domain (ZBTB20-ΔZnF) located at the C-terminus (S2 Fig) and examined its effect on dendritic spines and dendritic arborization (Fig 4A–4H). Truncation of the ZnF domains ablated the exclusively nuclear localization of ZBTB20 (Fig 4A and 4B). ZBTB20-ΔZnF induced significant reductions in dendritic spine area (*P* < 0.01) and spine length compared to ZBTB20-WT (Fig 4D). Spine breadth, bread-to-length ratio, and linear density were not significantly different between ZBTB20-ΔZnF and ZBTB20-WT (Fig 4D and 4E). This finding of altered spine size contrasted with the lack of effect on spine morphology of the analogous C-terminus variant, A620V (Fig 3J and 3K). This discrepancy of dendritic spine effects between ZBTB20-ΔZnF and the C-terminal variant, ZBTB20-A620V, may be due to mislocalization of ZBTB20-ΔZnF, potentially causing more dramatic effects on protein function.

Finally, we examined the role of the C-terminus in regulating dendritic morphology. ZBTB20-ΔZnF reduced apical arborization 225–350 μm from the soma, reduced apical dendritic length, and increased basal arborization at 75 μm from the soma, compared to ZBTB20-WT (Fig 4F, 4G and 4H). The effects of truncating the zinc finger domains mimicked the dendritic structural phenotypes of ZBTB20-A620V (Fig 3J and 3K) and emphasize an important role for the C-terminus of ZBTB20 in regulating dendritic structure.

## Discussion

*ZBTB20* gene variants have been identified in patients with NDDs and are thought to affect gene function, and thus neuronal function that is critical for learning, memory and behavior. A contribution of dosage imbalance of *ZBTB20* in a wide range of neurodevelopmental and neurobehavioral disorders has been suggested. Patients with heterozygous microdeletions, microduplications, or single nucleotide variants in different regions of the ZBTB20 protein show variability in clinical features that include autism, ID, and a more severe phenotype in patients with Primrose syndrome [12–17].

During murine embryonic development, the *Zbtb20* gene has been shown to play a role in regulating pyramidal neuron morphology and development and hippocampal connectivity [19, 21]. Furthermore, overexpresson of Zbtb20 in mice causes defects in cortical laminaton, and downregulation of *Zbtb20* expression affects maturation of CA1 pyramidal neurons, resulting in reduced apical dendritc arborization [18]. Here we confirmed the role of human ZBTB20 in regulating dendritic structure in pyramidal neurons. ZBTB20 isoforms showed distinct effects on dendritic spine morphology, and our data suggest the importance of the N-terminus of ZBTB20 in regulation of dendritic spine morphology. Studies of NDD-associated ZBTB20 variants further showed distinct effect on dendritic and synaptic structure from their wildtype counterparts. Furthermore, we found domain-specific effects of ZBTB20 variants, which differentially impact synaptic and dendritic structure, and may explain some heterogeneity of clinical features observed in patients with genetic variants located in different domains.

The P46R variant, which has been shown to be associated to ASD risk, altered dendritic spine size, suggesting a role for the N-terminus of ZBTB20 in selectively regulating synaptic structure. In contrast, the variant located in the central linker region (G346V) induced expansion of the basal dendritic compartment, and the variant in the C-terminal region (A620V) disrupted ZBTB20’s effects on apical dendritic elaboration. Interestingly, variants lacking the C-terminal zinc fingers domain ablated somal subcellular localization of ZBTB20, altered apical dendritic elaboration, and induced expansion of the basal dendritic compartment. It is speculated that modulating apical or basal dendritic arborization may have profound effects on the receptive field or the pool of available synaptic contacts of a particular neuron in a cortical circuit, and may contribute to altered connectivity in neurodevelopmental disorders [2, 25, 26]. As such, our future studies of the ZBTB20 variants analyzed here will focus on how their morphological effects alter the electrophysiological function of the neurons that express them.

Finding associations between distinct cellular phenotypes associated with genetic variants and distinct clinical phenotypes provides a hint for a possible mechanism by which distinct variants in the same protein might contribute to different disease states, potentially by disrupting neuronal structure or connectivity in a specific way (S3 Fig). Additional studies, perhaps involving RNA interference-mediated knockdown of ZBTB20 followed by an assessment of the variants’ ability to rescue the knockdown phenotype, would be required to further test this hypothesis. There are a number of proteins, such as NRXN1, NLGN4, SHANK3, SHANK2, and CNTNAP2, for which disease-associated rare variants have been associated with more than one disease state, such ASD and ID [27–30], and ZBTB20 similarly exerts pleiotropic effects.

Transcription factors are major regulators of dendritic morphology and the dendritic arbor, particularly in establishing cell type-specific dendritic branching patterns; however, most have been identified in *Drosophila*, while very few have been established as regulators of dendritic patterning in mammalian pyramidal neurons [2, 31]. Members of the BTB/POZ-ZF family of transcription factors, in particular, have been shown to regulate dendritic morphology: abrupt limits dendritic outgrowth in *Drosophila* and affects dendritic branching in a dose-dependent manner [23], and lola knockout leads to loss of restriction of projection neuron dendrites and defects in axon targeting [24]. A related family of proteins containing a BTB domain and kelch repeats also control dendritogenesis: NRP/B controls neurite outgrowth, an effect which requires the BTB domain [32]. Subtle changes in dendritic or synaptic structure can ultimately lead to enormous changes in information processing [25]. Dendritic arborization and dendritic spine morphology are key determinants of neuronal connectivity and function, and are disrupted in many NDDs, such as ASD and ID, in which behavioral and intellectual functions are affected [23, 26, 31]. Our results demonstrate that ZBTB20 affects the dendritic and synaptic structure of forebrain pyramidal neurons, genetic disruptions of which might play a role in phenotypic expression of NDDs. The data further point to the notion that molecules regulating synaptogenesis and neuronal connectivity are critical for normal cognitive and behavioral function.

## Materials and methods

### Expression constructs and antibodies

The *ZBTB20* open reading frame corresponding to the short and long isoforms was amplified from Human Fetal Brain MATCHMAKER cDNA library (Clontech, Mountain View, CA) and subcloned into a pcDNA3.1D/V5-His-TOPO vector which was tagged with V5 (short isoform) or GFP (long isoform) using a pcDNA 3.1 directional TOPO Expression Kit (Invitrogen, Carlsbad, CA). The primer sequences for the subcloning were: 5’-CACCATGCTAGAACGGAAGAAACC-3’ (long isoform, sense); 5’-CACCATGACCGAGCGCATTCACAG-3’ (short isoform, sense); and 5’-TCCGTCAGACACATGCATCC-3’ (common, antisense). The ZnF truncation construct was generated using the short isoform sense primer and an antisense primer with the sequence 5’-GCACAGCCAGTGGGCAAGGC-3’. ZBTB20 single nucleotide variant constructs were generated using QuikChange Site-Directed Mutagenesis Kit (Agilent Technologies, Santa Clara, CA) as described previously [33]. The primer sequences used for mutagenesis were: 5’-GCTGTTTTGTCTCCAGACCGAGCCCTCATCCACTCAACAC-3’ (P46R); 5’-GCAGAAGCCCCCGCTGAGGTTGGTCCGCAGACAAACCAG-3’ (G346V); and 5’- AGCAATGGGACCCCCCCTGTAGGCACACCCCCAGGTGCC-3’ (A620V). DNA from all constructs was sequence verified. The constructs were further verified using the TNT^®^ T7 Quick Coupled Transcription/Translation System. The proteins synthesized *in vitro* were then analyzed by SDS-PAGE. pEGFP-N2 and pmCherry-C1 (Clontech) were overexpressed in cultured neurons to outline the cells for morphometric analysis experiments. A polyclonal antibody against GFP was a generous gift from Dr. Richard Huganir (Johns Hopkins University). We purchased the following antibodies: GFP monoclonal (Millipore, Danvers, MA), V5 monoclonal (Invitrogen), and DsRed polyclonal (Clontech).

### Neuronal cultures

Dissociated cultures of primary cortical neurons were prepared from E18 Sprague-Dawley rat embryos (Charles River, Wilmington, MA) as previously described [34]. Neurons from 6–12 embryos per culture were transiently transfected as previously described at DIV 24–28 and allowed to express the transfected constructs for 3 days before fixation [34]. Studies were approved by Northwestern University’s Institutional Animal Care and Use Committee (IACUC) (Animal Study Protocol #2013–1939). Animals were singly housed in standard shoebox cages with Shepherd shacks and nestlets, on a 12:12 light-dark cycle, and given ad libitum access to food and water. Animals were monitored daily for signs of suffering or distress. If such signs were evident, animals were excluded from the study and euthanized via CO_2_ narcosis in accordance with Northwestern University’s IACUC guidelines and regulations under the approved protocol. Pregnant dams bearing embryos for cultures were also euthanized via CO_2_ narcosis in accordance with Northwestern University’s IACUC guidelines and regulations under the approved protocol.

### Immunofluorescent labeling

Cells were fixed in 4% formaldehyde/4% sucrose in PBS for 20 minutes. Fixed neurons were permeabilized and blocked simultaneously in PBS containing 2% normal goat serum and 0.1% Triton-X-100 for 1 hr at room temperature. Primary antibodies were added to PBS containing 2% normal goat serum overnight at 4°C, followed by 3 washes in PBS. Secondary antibodies were incubated in PBS with 2% normal goat serum for 1 hr at room temperature. Following 3 washes in PBS, coverslips were mounted onto slides using ProLong antifade reagent (Invitrogen).

### Visualization and quantification of dendrite and spine morphology

To examine dendritic spine morphology, neurons were transfected with GFP or mCherry + ZBTB20 constructs, fixed, visualized with an antibody against GFP or DsRed, and imaged and quantified as previously described [34]. Healthy pyramidal neurons were imaged using a confocal microscope (Zeiss LSM5 Pascal). *Z* stacks of images were taken using an oil-immersion 63x objective (NA = 1.4). Two-dimensional maximum projection images were reconstructed using Metamorph software (Molecular Devices, Sunnyvale, CA). Spine parameters (length, width, area and density) were measured in Metamorph. For each condition, 5–16 neurons were used, each from 3–4 separate experiments, and two dendritic branches (approximately 100 μm of linear distance) of each neuron were analyzed, resulting in analysis of 362–1206 spines per condition. Only spines on secondary and tertiary apical dendrites were measured to reduce variability. Neurons expressing point mutants or truncation mutants were compared only to ZBTB20-WT- and pcDNA-expressing neurons from sister cultures, transfected at the same time. Spine parameters for each condition were normalized to the GFP+pcDNA condition within each comparison, and represented as a relative percentage and relative s.e.m. To quantify dendritic morphology, pyramidal neurons expressing GFP or mCherry + ZBTB20 constructs were imaged using the 10x objective (NA = 0.17) on a Zeiss Axioplan2 upright microscope, and micrographs were acquired using a Zeiss AxioCam MRm CCD camera. Dendrites were traced in ImageJ to generate a binary image of the neuron’s total dendritic arbor. The apical dendritic arbor was identified as the distinctively thicker, longer, and more highly branched dendrite, and basal dendrites were identified as processes that radiated from the soma with shorter lengths and lower degree of branching. Images of the apical dendrites alone and basal dendrites alone were generated to allow for separate measurements of each dendritic compartment. The axon was identified by its distinctive morphology and was eliminated from quantification. Dendrite length was measured in MetaMorph to quantify total apical length and total basal length per neuron. To perform Sholl analysis, we utilized the Sholl analysis plugin for ImageJ (http://biology.ucsd.edu/labs/ghosh/software/) to measure the number of dendritic processes that cross concentric circles spaced 25 μm apart starting at the center of the soma. For dendritic length and Sholl analysis, 9–27 cells were measured each from 3–4 experiments, and the images were acquired and quantified by an experimenter blind to the conditions.

### Statistical analysis

For dendrite and dendritic spine morphology experiments, differences among condition means were identified by Student’s t tests performed in GraphPad and SPSS. Two-way repeated measures ANOVA with Bonferroni post tests were applied to Sholl analysis.

## Supporting information

S1 Table Raw values for quantification of dendritic spine areas, lengths, breadths, and breadth-to-length ratios. Boldface type indicates significant differences between point variant (or deletion variant) and wildtype counterpart.
(DOCX)

S1 Fig Dendritic spine numbers are not changed by overexpression of ZBTB20 variants. (A-C) Dendritic spine linear density (# of spines / 10 μm dendritic length) of neurons expressing pcDNA, ZBTB20-long-WT, ZBTB20-long-P46R, ZBTB20-WT, ZBTB20-G346V, or ZBTB20-A620V.
(DOCX)

S2 Fig Schematic of the ZBTB20 construct lacking the 5 zinc finger domains at the C-terminus of the protein. The BTB-domain (BTB), predicted sumoylation sites (blue boxes) in the central linker region are shown (see also Fig 1).
(DOCX)

S3 Fig Summary schematic of ZBTB20 variations and their corresponding cellular phenotypes. Dotted region at N-terminus of ZBTB20 protein indicates region present in ZBTB20-long-WT but not in ZBTB20-short isoform. The P46R variation of the long isoform was identified in patients with ASD and increases dendritic spine size, but has no effects on the dendritic arbor compared to its wildtype counterpart, ZBTB20-long-WT. The G346V variation of the short isoform was identified in patients with symptoms of both ASD and ID, and changes spine shape while enhancing the basal dendritic arbor (solid green lines indicate increased dendritic branching). The A620V variation of the short isoform was identified in patients with ID, and both enhances basal arborization (solid green lines) and reduces apical dendritic arborization (dotted red lines indicate lost dendritic branches) but has no effect on dendritic spines. The orange and blue wedges indicate the gradients of effects on dendrites and spines along the protein; each end of the protein appears to coincide with a more severe but single cellular phenotype, while the variation in the central region appears to induce mixed cellular effects.
(DOCX)
