# Supplementary material for: Neurodevelopmental disorder-associated ZBTB20 gene variants affect dendritic and synaptic structure
Source: PLoS One. 2018 Oct 3;13(10):e0203760. doi: 10.1371/journal.pone.0203760 (PMC6169859; doi:10.1371/journal.pone.0203760)
Supplement: S1 Table — Boldface type indicates significant differences between point variant (or deletion variant) and wildtype counterpart. (DOCX) [file pone.0203760.s001.docx]

**S1 Table**

| **Quantification of Dendritic Spine Morphology (mean ± s.e.m.)** | | | | |
| --- | --- | --- | --- | --- |
| Construct | Spine Area (µm^2^) | Spine Length (µm) | Spine Breadth (µm) | Breadth : Length |
| pcDNA | 0.6144 ± 0.0123 | 2.1314 ± 0.0360 | 0.7468 ± 0.0093 | 0.4013 ± 0.0066 |
| long-WT | 0.6221 ± 0.0096 | **1.9579 ± 0.0250** | 0.7696 ± 0.0068 | **0.4466 ± 0.0052** |
| long-P46R | **0.6524 ± 0.0118** | **2.0282 ± 0.0296** | 0.7820 ± 0.0084 | **0.4367 ± 0.0057** |
| pcDNA | 0.6264 ± 0.0099 | 2.1442 ± 0.0285 | 0.7608 ± 0.0076 | 0.4063 ± 0.0051 |
| WT | 0.6221 ± 0.0084 | 2.0934 ± 0.0253 | 0.7572 ± 0.0063 | 0.4158 0.0049 |
| G346V | 0.6274 ± 0.0113 | **1.9737 ± 0.0286** | 0.7795 ± 0.0085 | **0.1155 ± 0.0059** |
| pcDNA | 0.6264 ± 0.0099 | 2.1442 ± 0.0285 | 0.7608 ± 0.0076 | 0.4063 ± 0.0051 |
| WT | 0.6221 ± 0.0099 | 2.0934 ± 0.0253 | 0.7572 ± 0.0063 | 0.4158 0.0049 |
| A620V | 0.6269 ± 0.0094 | 2.1736 ± 0.0291 | 0.7632 ± 0.0071 | 0.4015 ± 0.0053 |
| pcDNA | 0.6238 ± 0.0097 | 2.1020 ± 0.0272 | 0.7611 ± 0.0076 | 0.4136 ± 0.0051 |
| WT | 0.6097 ± 0.0096 | 2.0436 ± 0.0282 | 0.7448 ± 0.0072 | 0.4181 ± 0.0056 |
| long-WT | 0.6452 ± 0.0110 | **1.9808 ± 0.0291** | **0.7859 ± 0.0079** | **0.4505 ± 0.0059** |
| pcDNA | 0.6473 ± 0.0147 | 2.0032 ± 0.0354 | 0.8029 ± 0.0109 | 0.4529 ± 0.0075 |
| WT | 0.6641 ± 0.0208 | 1.9710 ± 0.0457 | 0.8020 ± 0.0142 | 0.4632 ± 0.0102 |
| ΔZnF | **0.5989 ± 0.0140** | **1.8697 ± 0.0385** | 0.7688 ± 0.0102 | 0.4730 ± 0.0087 |
